# Cooperation and selfishness both occur during molecular evolution

**DOI:** 10.1186/s13062-014-0026-5

**Published:** 2014-11-26

**Authors:** David Penny

**Affiliations:** Institute of Fundamental Sciences, Massey University, Palmerston North, New Zealand

**Keywords:** Cooperation, Molecular evolution, Molecular level, Origin of life, Natural selection

## Abstract

Perhaps the ‘selfish’ aspect of evolution has been over-emphasised, and organisms considered as basically selfish. However, at the macromolecular level of genes and proteins the cooperative aspect of evolution is more obvious and balances this self-centred aspect. Thousands of proteins must function together in an integrated manner to use and to produce the many molecules necessary for a functioning cell. The macromolecules have no idea whether they are functioning cooperatively or competitively with other genes and gene products (such as proteins). The cell is a giant cooperative system of thousands of genes/proteins that function together, even if it has to simultaneously resist ‘parasites’. There are extensive examples of cooperative behavior among genes and proteins in both functioning cells and in the origin of life, so this cooperative nature, along with selfishness, must be considered part of normal evolution. The principles also apply to very large numbers of examples of ‘positive interactions’ between organisms, including both eukaryotes and akaryotes (prokaryotes). This does not negate in any way the ‘selfishness’ of genes – but macromolecules have no idea when they are helping, or hindering, other groups of macromolecules. We need to assert more strongly that genes, and gene products, function together as a cooperative unit.

**Reviewers:** This article was reviewed by Prof. Bill Martin (Düsseldorf), Dr. Nicolas Galtier (Montpellier) and Dr. Anthony Poole (Christchurch).

## Introduction

To over-simplify a little, it seems that for over a century the ‘selfish gene’ concept appears to have ruled – it has largely been assumed that evolution is in some sense ‘selfish’, and that consequently there is some ‘problem’ as to how cooperation might arise (reviewed in [1], who traces the selfishness idea back to the 19^th^ century). Perhaps it is better put as that the selfish aspect has been emphasized at the expense of the more cooperative aspects. For example, De Waal [2] p43 refers to it (rather critically) as ‘Veneer Theory’ – and sums it up by the phrase ‘scratch an altruist, and watch a hypocrite bleed’. Similarly, the subtitle of Ruse’s book [3] on the ‘Darwinian revolution’ is ‘Science red in tooth and claw’, and the title of Dawkins book [4] is ‘The selfish gene’.

But at the level of macromolecules (genes and proteins), a different perspective is also necessary. The molecules themselves do not know whether they are cooperating, or being selfish, and working together (cooperation) is a normal part of the functioning of the cell. In reality, the essential functional units of plants, animals and microbes are their genes and proteins, and these non-intelligent macromolecules simply have no idea whether or not they are cooperating with other genes and proteins. For example, an enzyme in the middle of a pathway does not ‘know’ whether or not it is essential for the production of a compound required by the organism, and whether it is also being regulated by other proteins on the pathway. Given the presence of the necessary cofactors and substrate the protein just carries out a reaction - quite oblivious of the fact that it is producing an essential intermediate. The enzyme is simply carrying out its reaction, and thereby creating a benefit to the organism – this issue has been a problem [5] in that it was not reasonably apparent to some researchers that benefits to the organism can be without any ‘cost’. So for macromolecules, if it works, it works, might be a better description.

Here we will consider primarily three levels of organisation – initially at the cellular level (of genes and proteins), then the level of the origin of life, and finally the level of organisms. It is particularly at these first two cellular/macromolecular levels where there are thousands of gene products working together that the cooperation of genes is the most obvious. At all three levels, and given this molecular approach, it is natural for both competitive and cooperative aspects of evolution to function. However, the principles are perhaps best seen at the level of macromolecules – both of genes and proteins. We will see later that this approach, where evolution is both cooperative and competitive is fully in agreement with the mechanistic approach to evolution. We will then come back to consider some of the earlier work in the Discussion.

## Review

### The level of genes and proteins (unicells)

We have already mentioned an enzyme in the middle of a pathway. The protein has no idea whether or not it is ‘essential’ for the production of some compound required by the organism; the proteins just carry out the reaction. However, the basic idea applies to all genes (and their proteins) in the cell. If a gene product is useful to the organism then there is a selective pressure for the maintenance of the gene. So if a mutant fails completely to give that functional protein, it is effectively a lethal mutation (at least in haploid cells). Conversely, if a protein is not necessary for the cell, then mutations will accumulate, or there may be selection for faster cell division in organisms with just a single center of DNA replication – giving selective pressure for loss of the gene [6].

The concept of non-intelligent molecules extends well beyond a single gene in the middle of a pathway. It is necessary for the organism to have a wide variety of small molecules, together with the genes and proteins that produce them. For example, most organisms require proteins that produce the 20 standard amino acids that are incorporated into proteins (unless they get some of them from their diet). The cell also requires enzymes to produce all the nucleotides that make up RNA and DNA; the bases and the sugars and the triose phosphates that are required for nucleotide incorporation. There must be enzymes for the production of other small molecules, energy, for protein and RNA and DNA biosynthesis, synthesis of tRNAs, regulation of the cell, production of essential cofactors, and so on. In reality, there are thousands of proteins involved in essential processes for most cells – it is estimated that some algae and plants have over 30,000 proteins [7], and some mutation in virtually any could be lethal. There are also many levels of regulation within cells, and there are proteins (chaperones) specialised for helping other proteins fold correctly. Similarly, it is little help to an organism if some enzymes function best at, say, <10°C, and others do not function at this temperature, but may need temperatures of >50°C to function effectively. In practice the cell is an integrated unit, oops, a cooperative system!

The main focus here has been on the level of genes and proteins as a functional system, but our previous example (optimal temperatures) helps lead to a related question of regulation. This is that there are many necessary modifications to proteins that allow these subcellular functions to work together (cooperate). At the level of regulation of proteins, there are many modifications to proteins, including methylation, phosphorylation, acetylation, etc., and any may lead to activation of a protein. There is also apoptosis (programmed cell death), when a cell (including a virally infected cell) literally self-destructs, rather than (for example) allowing the virus to mature and release thousands of descendent viruses. Certainly, viruses appear to be very old [8], and selfish elements have probably always existed. However, there are many interactions between proteins that lead to smaller subsets of proteins functioning together. For example, it has been known for 40 years that the small subunit of ribosomal RNA in *E.coli* combines with 21 ribosomal proteins, and that in vivo this occurs sequentially [9]. Only when the RNA/protein is fully integrated does it have maximum function. We also see similar specialization in subcellular organelles where there is compartmentation of many different functions, e.g. the mitochondria, and the nucleus.

So at this molecular/subcellular level, it is clear that we need to consider a living system as an integrated set of genes/proteins that work together as a functional/cooperative unit. If there are any unnecessary genes/proteins, then there will be no selection to maintain them. Similarly, any unnecessary genes/proteins are expected to result in the unit not reproducing so quickly and therefore there will be some selection against such systems (relative to those with no unnecessary genes [6]. We do have to be careful here because a ‘successful’ system will almost certainly have ‘defences’ against potential invaders – non-cooperative genes/proteins are also a potential part of any system.

So at this level of genes and proteins, it is clear that the macromolecules have no idea whether they are cooperating or not with other macromolecules, or whether they are interfering with other genes and their products. In practice, the functional cell has thousands of enzymes that have to work together. As an additional comment, it is not really useful just here to talk about multilevel selection because the general idea of macromolecules having no idea as to whether or not they are ‘cooperating’ applies at every level. It is a basic property of genetics of macromolecules that works at all levels of selection, even though the concept of multi-level selection can be important in many other contexts.

## The origin of life/Hyper-cycles

There has been good progress in understanding some of the principles behind the origin of life [10]. Early on Eigen and Schuster [11,12] introduced two concepts, the ‘quasi-species’ and the ‘hyper-cycle’ models, and both are going to be important for our discussion. The first was that there is expected to be a diversity of sequences around the optimal/’master’ sequence – this is the quasi-species model. The second concept was that, for example, molecule A copies molecule B, B copies C, C copies D and D copies A. Or, as Maynard Smith [13] puts it (perhaps a little facetiously), ‘God’ copies the molecule ‘save’, ‘save’ copies the molecule ‘the’, ‘the’ copies ‘queen’, and ‘queen’ copies ‘God’!

The first concept (quasi-species) was rapidly accepted, and is now standard [14]. It implies that the length of sequence (coding regions) that can be maintained by selection is set by the mutation rate. If the error-rate exceeds some threshold (which we call the ‘Eigen limit’ [15]) the system loses information, and essentially becomes randomised. Kun et al. [16] demonstrate that this should not be limiting for very early life, the accuracy is expected to be sufficient for early systems and some mutations are effectively neutral. Single-stranded RNA viruses (which have relatively high mutation rate about of around one mutation per 3000–10,000 nucleotides (often expressed as the number of mutations per replication [17]) and this limits RNA viruses to relatively shorter lengths. In practice, the influenza virus may have up to 8 separate RNA molecules [18], each well within the Eigen limit. Crotty et al. [19] report that the compound ribavirin that is an effective antagonist of several RNA viruses, and appears to work by increasing the mutation rate until it is above this ‘Eigen limit’ for the length of the RNA virus. At this increased mutation rate the RNA virus becomes randomised, and loses all information – it has entered the region of ‘error catastrophe’ which is the length beyond the Eigen limit. However, DNA polymerases have a much higher accuracy/fidelity (they can correct many errors by checking/correcting the new strand of DNA against the old (complementary/original) strand. Because of this higher accuracy, and in agreement with Eigen’s ideas, DNA viruses and DNA based organisms have very much larger genomes.

The second concept, hyper-cycles, is of more interest here because it emphasizes the ‘systems’ thinking that is more at the core of the current molecular approach to living systems, and it emphasizes molecules needing to ‘work together’. Initially the hyper-cycle concept may not have had the same consideration, but that is changing as the questions became more focused. Relatively early Boerlijst and Hogeweg [20] reported ‘game of life’ simulations (where spatial data was taken into account). Their simulations did allow ‘parasitic’ macromolecules – that would be readily copied by ‘active’ ribozymes, but the ‘parasite’ would not copy any other macromolecules. The parasitic molecules might become a dominant form locally, but because each molecule had a half-life, they would eventually break down. This meant that the ‘parasite’ died out in that local region, and so the existence of such ‘parasitism’ did not affect the long term evolution of the system. Adding this additional realism (a structured population without complete mixing, a half-life of the molecule, and allowing some non-functional molecules) meant that a stable and evolving system was possible. A recent example of a similar phenomenon with expanding populations and using yeast cells is reported in van Dyken et al. [21]. The concept of hyper-cycles is increasingly attracting attention of experimental systems. Much more recently Vaidya et al. [22] describe a system of just three RNA molecules which have an ability to evolve, and the authors show that such a system out-competes a ‘selfish’ system. Attwater and Hollinger [23] call it the ‘cooperative gene’.

On a related issue, Steel and Hordijk [24,25] have studied the mathematics of ‘autocatalytic sets’. It has been assumed for many years [26] that autocatalytic sets were basic – they are excellent at energy dissipation under steep gradients of chemical potential, such as are expected to occur in the origin of life. Hordijk and Steel [24,25] show that, even where it is random which molecules catalyse which reactions, that it is to be expected that there will be autocatalytic sets – they are an expected property of the system. Such cooperative systems are therefore ‘natural’, and are expected to occur.

Again, there is no assumption that all genes have to arise internally; there may be transfer between ‘organisms’. Carl Woese [27] has suggested that many organisms may have shared/swapped genes in the early stages of evolution. (Even in modern organisms there does seem to be transfer of mRNA between plants [28].) Again, there would have to be defence against universal uptake which might allow uptake of parasitic molecules, but the principle of the gene repertoire expanding to widen the combinations of genes is independent of this. Perhaps a similar conclusion may result from the widespread occurrence of sexual reproduction in eukaryotes [29]. It is expected that different combinations of genes might find a better ‘system’. The situation is possibly a little more complicated in Bacteria, but here ‘lateral gene transfer’ is more common, and the ‘bacterial’ species concept perhaps should be expanded to include the union of all the genes in sub-varieties/subspecies [30], not just the genes/proteins in a specific bacterial strain.

The results mentioned above of Boerlijst and Hogeweg [20] show that for realistic systems (not mixing universally) parasites can die out. Evolutionary mechanisms cannot distinguish between times to cooperate, and times to be selfish. At an early stage of evolution- which we might call the ‘molecular’ stage, the macromolecules have no idea if they are working cooperatively or selfishly. Again, all the same principles apply as for a living cell mentioned earlier.

The work of Eigen [11] and of Steel [24,25] that is referred to above is based on firm mathematics. So mathematically, there is considerable evidence to the effect that collaboration/cooperation works, that it is an essential part of a good strategy. Macromolecules really have no idea when they should, or should not, cooperate, and so in reality they are simply just non-intelligent macromolecules.

## Cooperation at the organismal level

Cooperation occurs in nature at all levels, not just at the level of genes/macromolecules. But perhaps we should start with the ‘multicellular’ question, which has been considered many times (e.g. [31,32]). The question was that some cells had to ‘give up’ the ability to reproduce when cell specialisation started to occur within many multicellular lineages. However, all the cells are considered to be virtually identical genetically, so inclusive fitness [33] should work well in this case [34]. Thus the issue may be more of a ‘how did multicellularity occur’, rather than a question of why did it occur. Certainly, we know that differential gene expression (being expressed in at different levels and in different tissues) occurs in all multicellular organisms – so this is another example of ‘molecules working together’ to achieve a better outcome.

Symbiosis is extremely common in nature [35], and indeed an interesting question might be to invert the question and find any organism where some symbiosis does not occur! Metagenomic studies of gut bacteria show that bacteria are an essential component of mammalian digestion – and therefore the bacteria have a good niche. For humans, it is becoming increasingly apparent that every individual has millions of commensal bacteria – and that they are essential for our digestion – we would not be able to survive and grow without these bacteria [36]. Bacteria themselves, though relatively simple, still gain from coordination and have direct contact between cells [37,38], and there is also significant amounts of transfer between adjacent *Myxobacteria* cells [39]. Some algae require surface bacteria that produce cobalamin (vitamin B12) that is required for their growth [40]. These bacteria can be grown by themselves, but fed a little algal extract and they secrete cobalamin. A survey found that 155 algae did not need vitamin B12, whilst 171 did [40]. However, land plants at least have developed new enzymes that bypass the need for cobalamin.

Most flowering plants have fungal mycorrhizae in their root system (see [41]), and where it appears that the fungi are better at taking up very low levels of phosphate, and are able to exchange this with the plant for sugars. Similarly, there are sometimes fungal associates in the leaves of grasses, and the fungi can produce two compounds that are toxic/distasteful to beetles and mammalian herbivores respectively. In this case, there is a veritable ‘industry’ [42] selecting fungal/plant associations where the fungi still produce the compounds harmful to beetles, but not the compounds that cause harm to mammalian herbivores (‘grass staggers’). Again there is an advantage to both the plant and the fungus. A similar case occurs in a marine sponge where a bacterial symbiont makes compounds that protect the sponge [43].

Lichens are an association (symbiosis) of algae and fungi [44]. They are extremely interesting from the ‘cooperative’ point of view in that they have formed many times during evolution. Lichens are certainly not a ‘one-off’, or rare, event. The fungal component can consist of any of 16 Orders of Ascomycete fungi, or two of Basidiomycetes, or two of Deuteromycetes [44]. Similarly the algal component can be cyanobacterial (a prokaryote, and which can also fix nitrogen as well as being photosynthetic) or a green or brown alga (both of which are eukaryotes). In lichens the cells (and genomes) are kept distinct, and occasionally three taxa may form one (a fungus, a green alga and a cyanobacterium). Similarly, nitrogen fixation comes up again when we find that a bacterium (*Rhizobium*) is associated with legume plants, and that the bacterium fixes nitrogen which the plant can use. However, there are many other nitrogen fixing associations between plants and bacteria [45].

There are also many other examples of endosymbiosis in eukaryotes. The origin of mitochondria by endosymbiosis is well known, and there are many examples of the transfer of (what originally were) mitochondrial genes to the nucleus of the host cell. However, the example of the origin of chloroplasts may be an even better example of the cooperative nature of some evolution in that there are many secondary endosymbioses – where a second eukaryote has taken up an algal cell (that already has a chloroplast, see [46]). A different example of endosymbiosis are bacteria occurring within insects, where the bacteria provide some essential amino acids for the insects [47], and possibly also supply cofactors (vitamins).

On a different level are other positive reactions. For example, birds flying long distances in V-formation gain from each other [48]. Similarly, cooperative breeding (where other birds help the nest) occurs in about 9% of avian species [49] – so that is over 800 avian species showing cooperative breeding. The authors report that the phenomenon is particularly common in Australia and sub-Saharan Africa – though less so in Europe and North America. A similar example are fish swimming and gaining speed from each other [50], the energy involved in swimming is less if there are other fish doing similar things.

Perhaps this section is unnecessary, and may get in the way of demonstrating cooperation at the macromolecular level. But these examples do show that some aspects of cooperation are extremely widespread in nature – we must consider evolution as both cooperative and selfish. There appear to be millions of cooperative interactions, even at the level of organisms, and this includes fungi, plants and animals. Indeed, we do not know of any species that is normally fully independent of all other species, though surely there are some, perhaps some Archaea or Bacteria in really extreme environments might give some examples. But such examples would be extreme, and it is certainly time to emphasise all aspects of evolution. So although we started at the level of gene sequences and proteins, and find that at that level cooperation is essential, it certainly does not stop there – cooperative systems occur at all levels in biology. But the primary interests here are the genes and gene products (especially proteins) that have to work together as a functional unit in order to be functional.

## Discussion

So yes, there is always the possibility of ‘cheats’ in evolution, and they include parasites and viruses. However, perhaps humans have a tendency of encouraging binary divisions (such as ‘parasites’ or ‘non-parasites’) whereas there is in reality a wide range (a spectrum) of alternatives. For example, a macromolecule might copy all other molecules, or copy most of them weakly, or copy just some of them weakly, or copy no others (but still be copied itself). So even here there is a big range of alternatives, and it is often not helpful to force binary choices [51]. Carlson et al. [52] point out that, from the view point of a relatively benign parasite (that does not kill the host) it is to this parasites disadvantage if the host becomes infected by an aggressive parasite that does kill the host. So it is to the first parasite’s advantage to prevent highly infectious, and potentially lethal, parasites. Even parasites have to be careful! Another alternative is that some insects, such as Harlequin ladybirds, carry microsporidia that are pathogenic to some other species of ladybird – but the Harlequin ladybird is resistant to that particular pathogen [53]. So, again, there are both positive and negative interactions, and they can vary in their effects on different species. But humans do seem to want binary choices?

In an important sense we agree with the many authors who have emphasized the mechanistic aspects of evolution, but such authors have often emphasized the basically ‘selfish’ nature of evolution (see the Introduction). However that is a part, but only a part, of the information available. We have recently emphasised [51,54] that Charles Darwin’s background included geology, and this appears to have been important in his accepting the need to study mechanisms – first in geology, and later in biology. So it is vital to search for mechanisms, and here we certainly agree with earlier authors. But equal attention must be paid to the cooperative nature of evolution, and this is perhaps more obvious at the molecular level?

More recently there has been more support for the cooperative aspect of evolution (see the dates on the references, and [55,56]) but perhaps there has been more emphasis on human cooperativity (and social insects), including the recent book [57]. There has been a concentration on the vital topic of the very high levels of cooperation between humans [58]. So the study of human cooperativity is certainly important, and supplements the present study, but cooperation is vital at all levels of evolution, starting with the genes and proteins. But the important point being emphasized here is that at the molecular genetic level of genes and proteins it is quite inappropriate (and erroneous) to attribute any intelligence to macromolecules.

Perhaps in the past we have emphasised the ‘cost’ of cooperation (see for example [59,60]), rather than the ‘gain’ from cooperation. In reality, there need be no necessary ‘cost’ to cooperation at the molecular level, but only gain. So cooperation does not necessarily incur any costs, only benefits – but this will vary, costs are also part of the system. From the point of view of macromolecules, there is a huge gain from working together – and the cellular system would not function at all if a very large group of macromolecules did not work together, that is, ‘cooperate’. Certainly, there will be times when there is a real ‘cost’ to some forms of cooperation, but that does not negate other times when there is no (or very little) cost. Again, humans wanting binary choices might be a problem.

There has been an increasing realisation of cooperation in evolution, perhaps dating back to Maynard Smith and Szathmary [61]) and more recently [62] have discussed the ‘major transitions in evolution’. However, at all stages there are going to be genes and proteins cooperating, and they these always include macromolecules. To some extent multi-level selection covers many of the issues here but we do want to emphasise that evolution is cooperative in that thousands of genes must always work together – as well as resisting ‘parasitic’ molecules. There is no evolution without the potential for cooperation!

## Conclusion

So overall the basic conclusion is quite simple at the molecular level. There must always be a very large number of macromolecules working together (cooperating) for any biological system to survive and to evolve, but simultaneously there must be defense against ‘cheats’. So genes and their products are not necessarily ‘selfish’, nor are they necessarily ‘cooperative’ – they simply do not know what is good for them. Could it be that genomes are basically ‘cooperative’, but no, that does not work as a concept either, they also composed of ‘unthinking macromolecules’. There is nothing unusual about macromolecules working together to make a functional system – it is quite ‘normal’ evolution.

## Reviewers’ report

### Reviewer 1: Professor Bill Martin, University of Duesseldorf, Germany

Report form: Here David Penny talks about cooperation between molecules cells and organisms. He concludes that ”There must always be a very large number of macromolecules working together (cooperating) for any biological system to survive and to evolve but simultaneously there must be defense against ‘cheats’. I submit that his conclusion is not new but the path he took to get there is so the essay is a worthwhile contribution to the record. I will not get into a debate with the author on the accuracy of all the statements. For example we knew about the importance and abundance of gut microbes long before metagenomics. Nor will I debate historical aspects for example the word symbiosis was coined by de Bary on the basis of studies on lichens which are often a symbiosis of prokaryotes and fungi not just algae and fungi as stated. Nor will I comment on attribution for example Szathmary and molecular parasites or Forterre and akaryotes. Happily Penny points out several interesting papers that have appeared of late on quite a few different topics. Few of us will have seen them all so this is another very positive aspect of the paper which offers so many opportunities to dig in and debate but life is so short.

Author’s response: *Yes, we seem to be basically on the same wavelength here. It is good that Martin points out that de Bary introduced the term ‘symbiosis’ to refer to the positive interactions in the lichens. Lichens are an important example of symbiosis/cooperation. The reference used does point out that ‘lichens’ include both eukaryote and prokaryote (akaryote) groups. Just 4 lines on this is spelled out in more detail. I guess I was using the term ‘algae’ in its archaic form that includes both eukaryotes and prokaryotes? However, the main point is that cooperative behaviour is ‘normal’, and is perhaps best illustrated at the molecular level.*

### Reviewer 2: Dr Nicolas Galtier, CNRS Montpellier, France

Report form: This is an interesting manuscript arguing that evolution is too often seen as driven by competitive forces cooperative processes being neglected. This argument is supported by the discussion of documented cooperative behaviours at all levels of biological organisation - molecules cells organisms species. I have two main comments.

First I found the very first sentences of the document very stimulating in large part because I can think of other colleagues that would presumably argue oppositely with similar conviction and eloquence. Penny says that “the selfish gene concept has [undully] ruled for over a century”. Others I think would rather suggest that the majority of the public and perhaps of biologists have on evolution an “intelligent design” perspective according to which biological structures and entities have been created in a way such that they work properly with each other. I guess the two viewpoints are justified depending on which community one refers to. Also I suggest that the personal philosophy psychology and social context of a scientist might influence his opinion regarding which of cooperation and competition is the dominant concept and whether this is deserved or not.

My second comment is about the series of interesting examples of cooperative behaviour that are considered and discussed throughout the manuscript. It seems to me that they don’t all reveal the same mechanisms and don’t equally challenge the main theory. Cooperation is unexpected between biological entities that reproduce independently from each other and compete for a common resource. Social insects, helper birds and apoptotic cells (evolutionary suicide) exemplify such paradoxical behaviours which are typically explained by the existence of genetic relatedness between the cooperating entities - they actually do not reproduce independently from each other. Some of the other examples discussed in the manuscript however concern entities that do not reproduce by themselves (molecules) or do not compete for the same resource (symbiotic species). This can be called cooperation from a human perspective in that we see them “working together” but this does not seem to be in conflict with the idea of competition-driven evolution - just like we usually don’t consider the fact that the various atoms of a biological molecule bind together as a challenge to the evolutionary theory. As discussed in the ms molecules cooperate because individuals in which they don’t are outcompeted by individuals in which they do. Cooperation and competition are not necessarily incompatible.

*Author’s response: I agree with much of what is written here. In regard to the first main point, my feeling is that the formal (mathematical?) analysis has concentrated much more on already having individuals – but that the cooperative nature of evolution is perhaps more obvious at the molecular level? Nevertheless, the principles of macromolecules not knowing whether they are cooperating or competing does hold at all levels, including of individuals. Again, and in agreement with Galtier’s second main comment, I certainly do not see any challenge to the ‘competition-driven’ evolution from the concepts presented here – and we must concentrate on mechanisms. It is necessary to include all microevolutionary processes in any concept of evolution.*

### Referee 3: Dr Anthony Poole, University of Canterbury, Christchurch, NZ

This interesting and timely manuscript by David Penny revisits and updates an important idea that is in need of a rethink, particularly given our increasingly detailed understanding of molecular systems.

The colloquial style of the manuscript hides the unification of some important concepts, but serves to make it very readable. In the spirit of the discussion format of peer review in Biology Direct, here are some points that may be worth further discussion.

I agree that we can productively view molecular systems as primarily cooperative, and my own understanding of the genetics literature on selfish genes is that these examples (primarily in the context of mendelian genetics, transposable element genetics and organellar inheritance in multicellular species) are the exceptions that allow us to see this cooperation. In this regard, the stochastic corrector model (Szathmáry & Demeter (1987) Group selection of early replicators and the origin of life. J. Theor. Biol. 128, 463–486; Szathmáry (2006) The origin of replicators and reproducers. Philos. Trans. R. Soc. Lond. B Biol. Sci. 361, 1761–1776) is a really important piece of work, and really should be discussed in this context. What is helpful about that model is that it shows how cooperativity can appear through selection at that higher level, even where there is competition between individuals at a lower level. It elegantly gets around the initial criticisms levelled at the hyper cycle by Maynard Smith (Ref [13]), and does so in a way that brings cells/compartmentalisation into the mix. Given Penny focuses on evolution from very early systems through to modern cellular systems, it could be helpful to include this alongside the hypercycle and autocatalytic sets work that is discussed.

Regarding endosymbiosis, Maynard Smith wrote a very interesting piece, hidden away in a book chapter (Maynard Smith, J. (1991) A Darwinian view of symbiosis. In: Symbiosis as a Source of Evolutionary Innovation (pp. 26–39). Cambridge, MA: MIT Press), that explains how compartmentalisation is critical to evolutionarily stable mutualisms. A lot of this was of course further developed in Maynard Smith and Szathmary’s The Major Transitions in Evolution (Ref [61]), but the book chapter is particularly clear on this. To my mind, the importance of compartmentalisation in evolutionary transitions really helps to cement the main thesis of the present manuscript, that cooperation is central to understanding biological systems at the molecular level. This also helps in terms of drawing the line between the evolution of interactions critical to the emergence of multicellularity or endosymbioses, relative to ecological interactions, where, as Penny notes, it would indeed be difficult to find any organism where symbiosis does not occur. Thompson has made that point with regard to ecological interactions, with the following thought experiment: “Try to imagine a plant that can survive and reproduce in a real ecosystem without using, in addition to its nuclear genome, most of the following: a mitochondrial genome (to convert energy); a chloroplast genome (to regulate photosynthesis); one or more mycorrhizal fungal genomes (to improve nutrient and water uptake); the genomes of pollinators (to assist in reproduction); and the genomes of a few birds, mammals, or ants (to move seeds around the ecosystem). Each plant is part of a complex web of interacting mutualists.”

(Thompson (2006) Mutualistic webs of species. Science 312, 372–373) Regarding the final paragraph before the discussion, where Penny states that ‘Perhaps this section is unnecessary,’ I do agree that it is not needed to appreciate ‘cooperation at the macromolecular level’, but I think it is helpful to have made the comments on broader interactions. For one, this broader issue of macromolecular cooperation is something that will have to be discussed within the context of how genomics might be utilised in the study of ecosystem interactions (Poole et al. (2012) ‘Ecosystomics’: Ecology by sequencer. Trends. Ecol. Evol. 27, 309–310). With that in mind, a clear statement of the importance of compartmentalisation for understanding ‘macromolecular cooperation’ may prove to be important - Dagg has for example written on the implications of the selfish gene and the extended phenotype for understanding how the atmosphere is a by-product not a product of selection (Dagg (2002) Unconventional bed mates: Gaia and the selfish gene. Oikos 96, 182–186).

Compartmentalisation may be an important delineator, since moving towards ecosystem interactions, it is important to avoid agency, such as incorrectly invoked in the Gaia model or through misinterpretation of the biology underlying the phrase ‘the selfish gene’. So in that regard, making the statements that Penny does regarding molecules not ‘knowing’ they are cooperating or being selfish, while seemingly obvious, are important because scientists do sometimes fall into the trap of ascribing agency (e.g. Gaia) or pan selection (e.g. early life as a megaorganism of cooperating genes) to systems.

*Poole suggests expanding the discussion even more, and I agree that might be useful, but I have kept the paper the same to concentrate on the principles. I agree that the ‘exceptions’ (such as horizontal gene transfer) allow us to see even better the principles of cooperativity that are essential for any living system as we know them. I have deliberatively kept out of the ‘group selection’ argument because it tends to divide people from the essential issues discussed here. Again, perhaps the compartmentalisation reasoning would help even further. It certainly would be difficult to find any organism that does not interact with others, and some of Poole’s examples are very useful. Many of his references are important for the general issues discussed here. I have been through the manuscript checking the suggestions/typos that Poole has picked up - I hope they have now all been corrected.*

## Abbreviation

‘Eigen limit’: The length of RNA or DNA that can be maintained for a specified mutation rate
